# DNA methylation changes in the postmortem dorsolateral prefrontal cortex of patients with schizophrenia

**DOI:** 10.3389/fgene.2014.00280

**Published:** 2014-08-26

**Authors:** Shusuke Numata, Tianzhang Ye, Mary Herman, Barbara K. Lipska

**Affiliations:** ^1^Human Brain Collection Core, National Institute of Mental Health, National Institutes of HealthBethesda, MD, USA; ^2^Department of Psychiatry, Course of Integrated Brain Sciences, Medical Informatics, Institute of Health Biosciences, The University of TokushimaTokushima, Japan; ^3^The Lieber Institute for Brain Development, Johns Hopkins University Medical CenterBaltimore, MD, USA

**Keywords:** schizophrenia, DNA methylation, SNP, postmortem, array, expression

## Abstract

**Background**: Schizophrenia is a complex psychiatric disorder with a lifetime morbidity rate of 0.5–1.0%. The pathophysiology of schizophrenia still remains obscure. Accumulating evidence indicates that DNA methylation, which is the addition of a methyl group to the cytosine in a CpG dinucleotide, might play an important role in the pathogenesis of schizophrenia.

**Methods:** To gain further insight into the molecular mechanisms underlying schizophrenia, a genome-wide DNA methylation profiling (27,578 CpG dinucleotides spanning 14,495 genes) of the human dorsolateral prefrontal cortex (DLPFC) was conducted in a large cohort (*n* = 216) of well characterized specimens from individuals with schizophrenia and non-psychiatric controls, combined with an analysis of genetic variance at ~880,000 SNPs.

**Results:** Aberrant DNA methylation in schizophrenia was identified at 107 CpG sites at 5% Bonferroni correction (*p* < 1.99 × 10^−6^). Of these significantly altered sites, hyper-DNA methylation was observed at 79 sites (73.8%), mostly in the CpG islands (CGIs) and in the regions flanking CGIs (CGI: 31 sites; CGI shore: 35 sites; CGI shelf: 3 sites). Furthermore, a large number of cis-methylation quantitative trait loci (mQTL) were identified, including associations with risk SNPs implicated in schizophrenia.

**Conclusions:** These results suggest that altered DNA methylation might be involved in the pathophysiology and/or treatment of schizophrenia, and that a combination of epigenetic and genetic approaches will be useful to understanding the molecular mechanism of this complex disorder.

## Introduction

Schizophrenia is a complex psychiatric disorder with a lifetime morbidity rate of 0.5–1.0%. Accumulating evidence indicates that DNA methylation, which is the addition of a methyl group to the cytosine in a CpG dinucleotide, might play an important role in the pathogenesis of schizophrenia. For example, L-methionine, a precursor of S-adenosylmethionine, which donates its methyl group to various acceptors, exacerbates the psychotic symptoms of schizophrenia patients (Pollin et al., 1961; Cohen et al., 1974). L-methionine-treated mice exhibited increased DNA methylation that was accompanied by decreased mRNA levels of specific genes, and by behavioral changes similar to those seen in schizophrenia (Tremolizzo et al., 2002, 2005). In addition, an increased mRNA expression of DNA methyl-transferases (*DNMT1* and *DNMT3a*) has been observed in schizophrenia (Veldic et al., 2004, 2005; Ruzicka et al., 2007; Zhubi et al., 2009). Furthermore, aberrant DNA methylation in brains of patients with schizophrenia (Abdolmaleky et al., 2005, 2006, 2011; Grayson et al., 2005; Iwamoto et al., 2005; Tamura et al., 2007; Mill et al., 2008; Tolosa et al., 2010; Wockner et al., 2014) and the associations of different DNA methylation patterns with phenotypic discordance of schizophrenia between twins (Petronis et al., 2003; Dempster et al., 2011; Kinoshita et al., 2013) have been reported. However, the sample sizes in these previous epigenetic studies of schizophrenia were relatively small and the number of CpG sites interrogated was limited.

Tissue-specific differences in DNA methylation have been extensively documented (Christensen et al., 2009; Davies et al., 2012). Thus, since schizophrenia is a brain disorder, it is particularly important to examine the epigenetic modifications of the brains in patients with schizophrenia, rather than in the peripheral tissues. In this study, we conducted a genome-wide DNA methylation profiling (27,578 CpG dinucleotides spanning 14,495 genes) of the dorsolateral prefrontal cortex (DLPFC), a brain region implicated in cognition and schizophrenia, in a large cohort of well characterized specimens (106 patients with schizophrenia and 110 non-psychiatric controls), and identified genes whose methylation levels differed between patients with schizophrenia and controls. We also conducted a genome-wide association analysis of single nucleotide polymorphisms (SNPs) with DNA methylation in the same samples, and revealed a large number of cis-methylation quantitative trait loci (mQTL), including associations with risk SNPs implicated in schizophrenia. These results will add further insight into the molecular mechanisms of the pathophysiology of schizophrenia.

## Materials and methods

### Human postmortem brain tissue collection

Postmortem human brains (*n* = 185) were collected through the Offices of the Chief Medical Examiners of Washington, DC and Virginia, Northern District by the Section on Neuropathology at the Clinical Brain Disorders Branch, National Institute of Mental Health, National Institutes of Health (NIH), according to the NIH Institutional Review Board (IRB) and ethical guidelines under protocol #90-M-0142. Thirty one additional postmortem human brain specimens were collected through the Stanley Medical Research Institute. Clinical characterization, neuropathological screening, toxicological analyses, and dissections of the DLPFC were performed as previously described (Lipska et al., 2006). Briefly, all patients met DSM-IV criteria for a lifetime Axis I diagnosis of schizophrenia (*n* = 97) or schizoaffective disorder (*n* = 9) according to DSM-IV, and controls were defined as those individuals with no history of significant psychological problems or psychological care, psychiatric admissions, or drug detoxification and with no known history of psychiatric symptoms or substance abuse, as determined by both telephone screening and medical examiner documentation as well as negative toxicology results. Demographic data for these samples are summarized in Supplementary Table S1.

### Genotyping methods

SNP genotyping with Human1M-Duo V3 BeadChips (Illumina Inc., San Diego, CA) was carried out according to the manufacturer's instructions, using DNA extracted from cerebellar tissue. Genotype data were analyzed using the Genotyping Analysis Module within the BeadStudio software (Illumina Inc.). For data analysis, 875,511 SNPs with missing calls <2%, Hardy–Weinberg equilibrium *p*-values ≥0.001, and minor allele frequencies ≥0.015 were used, from among a total of 1,199,187 SNPs.

### Methylation methods

Genomic DNA was extracted from 100 mg of pulverized DLPFC tissue using the phenol-chloroform method. Bisulfite conversion of 600 ng genomic DNA was performed using the EZ DNA methylation kit (Zymo Research). Methylation of DNA extracted from the DLPFC was assessed according to the manufacturer's instructions using Infinium HumanMethylation27 BeadChips (Illumina Inc.). Quantitative measurements of DNA methylation were determined for 27,578 CpG dinucleotides spanning 14,495 genes. CpG sites were selected by Illumina Inc. in the gene promoter regions, within 1 kb upstream and 500 bases downstream of the transcription start sites (TSSs). CGIs, CGI shores (0–2 kb from CGIs), CGI shelves (2–4 kb from CGIs) were defined as in a previous paper (Irizarry et al., 2009). DNA methylation data were analyzed using the Methylation Analysis Module within the BeadStudio software (Illumina Inc.). Normalization was carried out using lumiMethyN function from lumi package. The DNA methylation level of each CpG site was calculated as an *M*-value, which is the log2 ratio of methylated and unmethylated probe intensities. The technical schemes of this array have been described in detail in a previously published paper (Bibikova et al., 2009). Qualified CpG sites used in statistical analyses were defined as follows: (1) detected in 80% subjects, (2) excluded sex chromosome, (3) excluded 100% non-specific probes that completely match to other sequences, (4) excluded probes with SNPs at the CpG site with minor allele frequency MAF > 0.1%. A list of potential non-specific probes and polymorphic probes of Illumina Human 27K Methylation Array can be downloaded at http://braincloud.jhmi.edu/downloads.htm. The final data set included 25,156 CpG sites (Supplementary Table S2). To ensure data reproducibility, 10 samples were analyzed in duplicates starting from the bisulfite conversion step, and high reproducibility was observed (*r*^2^ ranged from 0.9973 to 0.9921). For validation, we used 92 samples from the current study and measured methylation status at 34 CpG sites using Illumina custom GoldenGate platform. The CpG site positions for the probes were exactly the same as in the Infinium arrays. The correlation between the data from the two platforms was very high (*r*^2^ = 0.79) as described in Numata et al. (2012).

### Statistical methods

Surrogate variable analysis was used to account for known and unknown factors affecting the data, including batch effects (Leek and Storey, 2007). A general linear model was then used to examine the effects of the primary variables: age, sex, race, and diagnosis as well as the surrogate variables. The residuals from multiple regression analysis were used to analyze associations with SNP genotypes by PLINK (Purcell et al., 2007). Outliers were identified using Grubb's test and removed from further analysis. SNPs within 1 Mb of a CpG site were defined as cis-SNPs, as in previous studies (Gibbs et al., 2010; Zhang et al., 2010; Numata et al., 2012). In these analyses, Bonferroni correction for multiple testing was applied at the 0.05 level.

### Transcription methods

For correlations between DNA methylation and expression, we utilized previously published expression data from the DLPFC obtained using Human HT-12_V3 Illumina BeadArrays as described in detail in Ye et al. (2012). Only probes expressed above the background (*p* < 0.05) in at least 80% of subjects were analyzed. Normalization was carried out using lumi R package. The ComBat R package was used for batch effects. Surrogate variable analysis was used with SVA R package with age, sex, race, and diagnosis as primary variables. A step-wise model selection was used for each gene, and a multiple linear regression analysis was performed with the best fit model.

## Results

### Diagnostic differences in DNA methylation

**Figure 1:**
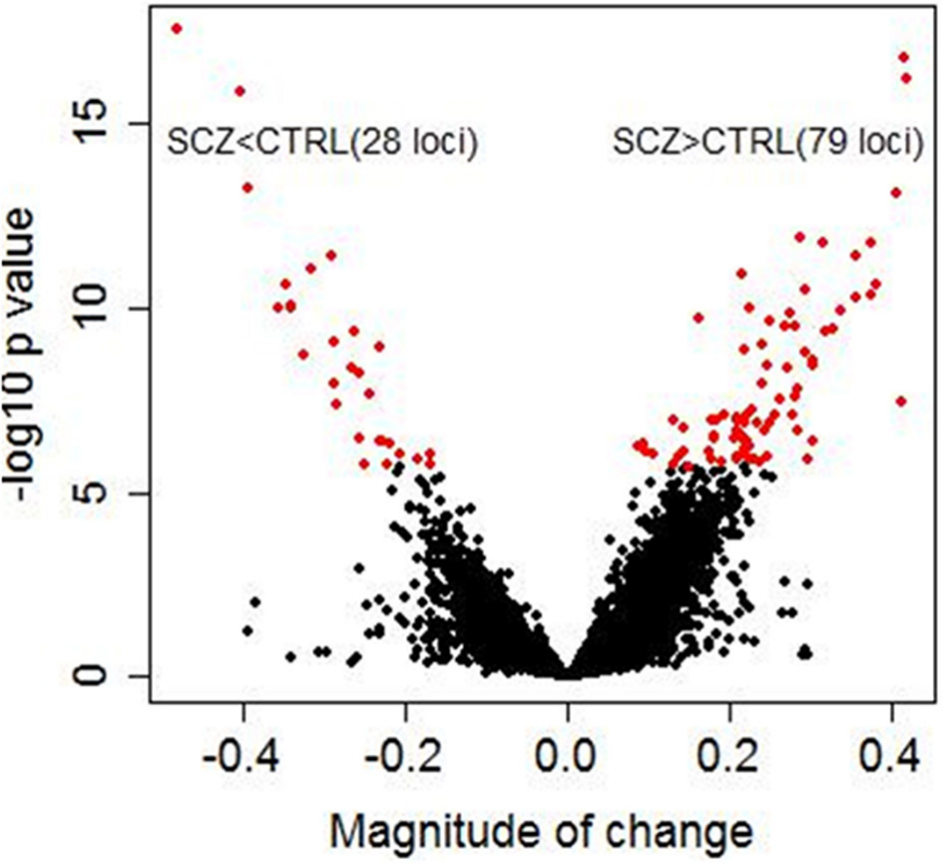
**Diagnostic differences in DNA methylation.** The x axis represents magnitude of a change (beta value) between controls and schizophrenia subjects calculated by linear regression analysis in 216 samples. The y axis represents −log 10 *p*-values. Each dot represents an individual CpG site (a total of 25,156 CpG sites). Red dots represent 107 CpG sites that showed significant diagnostic differences between schizophrenic subjects and controls (5% Bonferroni correction). Magnitude of change >0 corresponds to higher methylation in patients with schizophrenia than in controls, whereas magnitude of change <0 corresponds to lower methylation in patients with schizophrenia than in controls. Of these 107 CpG sites, 79 sites (73.8%) demonstrated higher methylation in patients with schizophrenia than in controls.

**Figure 2:**
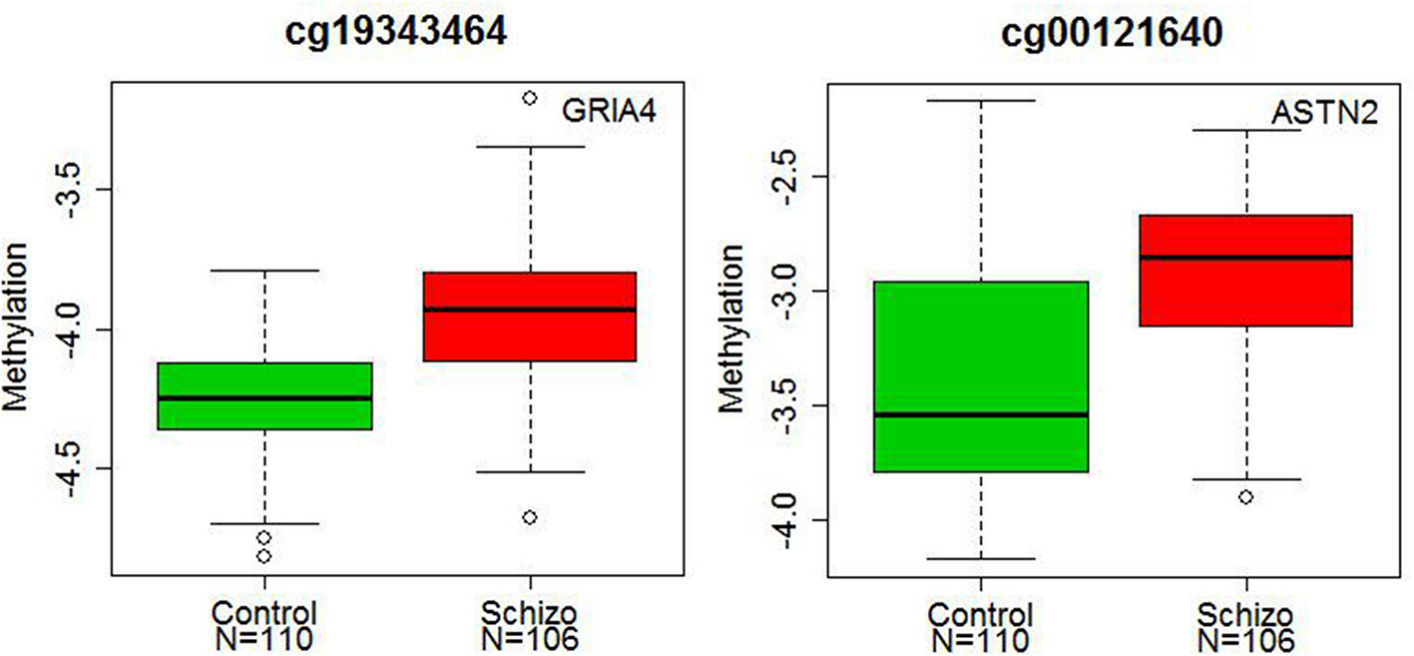
**Diagnostic differences in the DNA methylation levels of *GRIA4* and *ASTN2*.** The x axis represents diagnostic groups (control and schizophrenia), and the y axis represents DNA methylation levels (log2 ratio of methylated and unmethylated probe intensities). The lines in the bar graph indicate the median, and the bars (whiskers) represent the minimum and maximum values after the removal of the outliers. The CpG sites of the *GRIA4* (cg19343464) and *ASTN2* (cg00121640) showed significantly higher DNA methylation in patients with schizophrenia than in controls (*p* = 3.55 × 10^−7^ and *p* = 1.09 × 10^−6^).

Significant diagnostic differences in DNA methylation were observed at 107 CpG sites at 5 % Bonferroni correction (*p* < 1.99 × 10^−6^, Supplementary Table S3) out of a total 25,156 CpG sites examined. Of these 107 CpG sites, 79 sites (73.8%) demonstrated higher DNA methylation in schizophrenia than in controls (Figure 1). Examples of diagnostic-biased genes include *GRIA4* (glutamate receptor, inotropic, AMPA4) and *ASTN2* (astrotactin 2) (Figure 2), both of these genes have been previously implicated in schizophrenia (Makino et al., 2003; Vrijenhoek et al., 2008; Wang et al., 2010). When the 79 sites hypermethylated in schizophrenia were classified into four categories (CGI, CGI shore, CGI shelf, and others), most of them (87.3%) were located in the CGIs and in the regions flanking CGIs (CGI: 31 sites; CGI shore: 35 sites; CGI shelf: 3 sites; others: 9 sites). In contrast, when 28 sites, which demonstrated significantly reduced DNA methylation in schizophrenia, were classified into the same four categories, most of them (85.7%) were located not in or near CGIs but outside CGIs (i.e., others: 23 sites; CGI: 1 site; CGI shore: 3 sites; CGI shelf: 1). There was no effect of smoking on DNA methylation in any group.

### The mQTL analysis

A genome-wide association analysis of SNPs with DNA methylation was conducted in the combined cohort of controls and cases to identify mQTL, as has been done in other previous QTL studies (Liu et al., 2010; Zhang et al., 2010; Gamazon et al., 2013). In the cis-analysis (cis defined as within 1 Mb of a CpG site), 36,366 SNP-CpG pairs were significantly correlated (Bonferroni corrected *p* < 0.05) (Supplementary Table S4). When the association analysis was performed in the subjects with schizophrenia and controls separately, 89.7% of cis-associations from the subjects with schizophrenia and 89.5% of cis-associations from controls were found in the analysis of the combined cohort (Supplementary Table S5).

Among these significant cis-associations in the combined cohort, we found SNPs that have been previously associated with increased risk for schizophrenia. For instance, as shown in the recent study (Smith et al., 2013), a frequent promoter variant rs6311 of the *HTR2A* [5-hydroxytryptamine (serotonin) receptor 2A] gene, widely implicated in human neuropsychiatric disorders and significant in the meta analysis in the Schizophrenia Gene database (Allen et al., 2008), decreases usage of an upstream transcription site encoding a longer 5′UTR with greater translation efficiency. Our data show that the A allele, which increases risk for schizophrenia, is associated with higher methylation at the HTR2A locus cg00308665 (Bonferroni corrected *p* = 3.02 × 10^−16^). These data suggest a possible interplay between higher CpG methylation and repressed expression of the extended 5′UTR and might offer a molecular mechanism for the statistical association of rs6311 with mental disorders.

**Figure 3:**
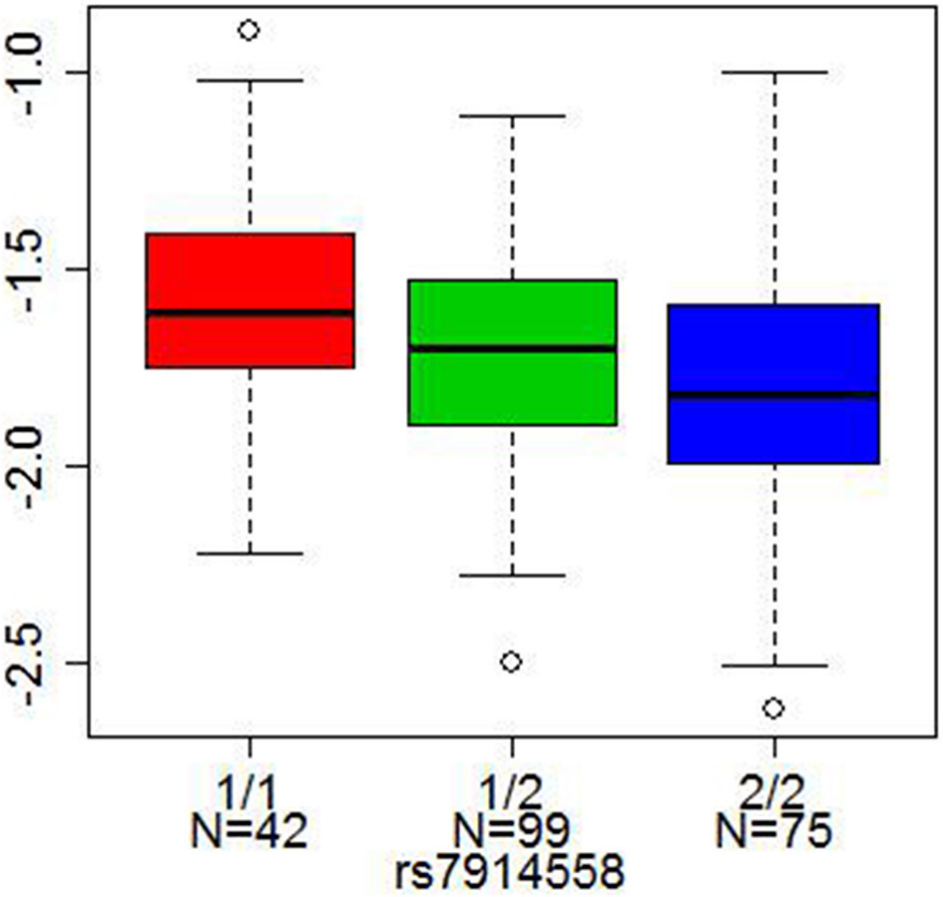
**Cis-association of a SNP with DNA methylation.** The x axis represents three genotypes of each SNP (1/1 red, 1/2 green, and 2/2 blue, where 1 = A or T allele and 2 = G or C allele), and the y axis represents DNA methylation levels. The lines in the bar graph indicate the median, and the bars (whiskers) represent the minimum and maximum values after the removal of the outliers. rs7914558 was associated with DNA methylation status at cg15439196 in the *CNNM2* gene (Bonferroni corrected *p* = 1.74 × 10^−2^).

Two other SNPs, rs13219354 (*PRSS16*) and rs3747600 (*C16orf5*), with significant mQTLs in our analysis, were found among the top 18 markers in another large genome-wide association study of schizophrenia (a total of 12,945 cases and 34,591 controls) by Stefansson et al. (2009). One SNP, rs7914558 (*CNNM2*), which was significant after Bonferroni correction in our analysis, appeared among the top 10 markers in the mega-analysis combining genome-wide association study data by the Schizophrenia Psychiatric Gnome-Wide Association Study Consortium (2011) (a total of 17,836 cases and 33,859 controls). This intronic SNP in *CNNM2* (rs7914558) predicted DNA methylation status at the cg15439196 CpG site in *CNNM2* and at cg00035347 site of the neighboring gene, *NT5C2* (5′-nucleotidase, cytosolic II), Bonferroni corrected *p* = 1.74 × 10^−2^ and 6.05 × 10^−22^, respectively), Figure 3.

### DNA methylation correlates with expression of a subset of genes

To interrogate relationships between DNA methylation and transcription, we correlated (Pearson's correlation) methylation status at CpG loci in gene promoters with expression of all probes on the array corresponding to the same gene using genome-wide transcriptional profiling data of the DLPFC from the same subjects (Ye et al., 2012) (all pairs were within 100 kb, a total number of such correlations was 107,031). Overall, across all CpG sites, there was poor correlation with expression. We identified only 86 correlations significant after Bonferroni correction (*p* < 4.63 × 10^−7^), of which 73 (84.9%) were negative (Supplementary Table S6). Of particular interest is *GNMT* (glycine N-methyltransferase) with high negative correlation between DNA methylation and expression (*r* = −0.42, *p* = 4.5 × 10^−10^). *GNMT* catalyzes the synthesis of sarcosine from glycine using S-adenosylmethionine as the methyl-donor in one carbon metabolic pathway, and Gnmt^−/−^ mice have shown dysregulation of DNA methyltransferases (*DNMT1* and *Dnmt3a*), ErbB (*Nrg1* and *ErbB4*), and mTOR (*Akt2, S6, S6k1*, and *S6k2*) in the cortex, as well as abnormal behaviors associated with schizophrenia (Yang et al., 2012).

## Discussion

We identified over one hundred CpG sites with aberrant DNA methylation in schizophrenia in this genome-wide DNA methylation profiling study performed in a large cohort of 106 patients with schizophrenia and 110 non-psychiatric controls. To date, 11 DNA methylation studies were conducted on schizophrenia using postmortem brains, but the sample sizes of these studies were relatively small (~35 patients with schizophrenia) (Abdolmaleky et al., 2005, 2006, 2011; Grayson et al., 2005; Iwamoto et al., 2005; Dempster et al., 2006; Tamura et al., 2007; Mill et al., 2008; Tochigi et al., 2008; Tolosa et al., 2010; Wockner et al., 2014).

Our study demonstrated that altered DNA methylation in schizophrenia was more likely to show a pattern of hyper- DNA methylation, and that it occurred at CpG sites not only in the CGIs but also in the CGI shores. These findings are in agreement with a recent genome-wide DNA methylation study using the leukocytes of patients with schizophrenia (Kinoshita et al., 2013). In line with our results, an increased mRNA expressions of DNA methyl-transferases has been found in post-mortem brains of schizophrenia (Veldic et al., 2004, 2005; Ruzicka et al., 2007; Zhubi et al., 2009). We identified a number of genes with significant epigenetic alterations in schizophrenia, and some of these genes, such as *GRIA4, ASTN2*, and *DCDC2* (doublecortin domain containing 2) with increased DNA methylation at particular CpG loci, have previously been implicated in schizophrenia. For example, genetic variations *in GRIA4*, a subunit of AMPA receptor that mediates fast synaptic excitatory neurotransmission, have been associated with schizophrenia and antipsychotic responses in patients. Moreover, *GRIA4*-deficient mice exhibit schizophrenia-related phenotypes (Makino et al., 2003; Lavedan et al., 2009; Sagata et al., 2010; Fijal et al., 2012). *ASTN2* is expressed at high levels in migrating cerebellar granule neurons at developmental stages when glial-guided migration occurs (Wilson et al., 2010). SNPs in this gene have been associated with schizophrenia and metabolic side effects of antipsychotic drugs, as well as with autism, attention deficit hyperactivity disorder, hippocampal volume, and cognition (Lesch et al., 2008; Vrijenhoek et al., 2008; Glessner et al., 2009; Wang et al., 2010; Adkins et al., 2011; Lionel et al., 2011; Bis et al., 2012). *DCDC2* gene is located on chromosome 6p22.1, a region with strong evidence of linkage with schizophrenia (Shi et al., 2009). This gene has been also identified as a candidate gene for reading disability, and implicated in neuronal migration (Meng et al., 2005). SNPs in this gene have been associated with cortical gray matter and resting state fMRI activity in language-related brain regions in patients with schizophrenia (Jamadar et al., 2011, 2013).

We did not find changes in methylation status for a number of genes reported in the previous postmortem brain studies of DNA methylation based on candidate gene approaches. For instance hyper- DNA methylation of *RELN, SOX10* [SRY (sex determining region Y)-box 10], *FOXP2* (forkhead box P2), and *HTR2A* as well as hypo- DNA methylation of *MB-COMT* (membrane-bound catechol-O-methyltransferase) and *HTR2A* have been reported in schizophrenia (Grayson et al., 2005; Iwamoto et al., 2005; Abdolmaleky et al., 2006, 2011; Tolosa et al., 2010). Although Infinium HumanMethylation27 BeadChips covered these five genes, the exact locations of CpG sites were different from those in the previous studies. This may explain discrepancies between our results and those in the previous studies. When we compared our data with the previous genome-wide DNA methylation study using CpG-island microarrays (Mill et al., 2008), we found one common gene, *MRPS14* (mitochondrial ribosomal protein S14), which showed significantly higher- DNA methylation changes in schizophrenia in both studies. This result suggests that there may be changes in mitochondrial function in schizophrenia (Anglin et al., 2012). Recently, Wockner et al. identified 4641 probes corresponding to 2929 genes which were found to be differentially methylated between diagnosis in frontal cortex post-mortem brain tissue from 24 patients with schizophrenia and 24 unaffected controls using Illumina Infinium HumanMethylation450 BeadChip (Wockner et al., 2014). Of 4641 probes they identified, 198 common probes were used between Infinium HumanMethylation27 BeadChip and Infinium HumanMethylation450 BeadChip. Of 198 probes, we found one common probe between studies in the *FAM5C* (family with sequence similarity 5, member C) gene, also known as *BRINP3*, which showed significantly higher- DNA methylation changes in schizophrenia in both studies. *BRINP* family is developmentally regulated neural-specific proteins (Kawano et al., 2004), and *BRINP3* is related to proliferation and migration (Shorts-Cary et al., 2007).

Finally, this study revealed a large number of cis-mQTLs in the human prefrontal cortex, including risk SNPs associated with schizophrenia in recent genome-wide association studies (Stefansson et al., 2009; Schizophrenia Psychiatric Gnome-Wide Association Study Consortium, 2011). This data set can be useful to reveal potential roles of previously identified risk SNPs associated with schizophrenia as well as discover novel target genes in schizophrenia.

There are several limitations to the present study. First, the coverage of transcriptome by mRNA expression probes and the coverage of methylome by CpG site probes were limited in this study. Especially, more comprehensive methylome mapping, including CpG sites outside CpG islands and intragenic regions will be needed to do a broader analysis of DNA methylome in schizophrenia. We have already initiated such a study. Second, we used genomic DNA extracted from the heterogeneous mixture of various cell types. However, cell type-specific variations in DNA methylation have been documented (Iwamoto et al., 2011) and thus cell type-specific methylome mapping should be performed. Third, we did not investigate histone modifications which are also known to play a role in regulating gene expression (Berger, 2007). Moreover, it is not possible to differentiate methylation from 5-hydroxymethylation of cytosine which also plays a critical role in gene regulation (Bhutani et al., 2011). Finally, it is possible that despite our efforts to remove confounding variables, batch effects and other factors, including smoking and body mass index (Breitling et al., 2011; Dick et al., 2014) might have contributed to the observed differences between the diagnostic groups.

Despite these limitations, our results suggest that altered DNA methylation might be involved in the pathophysiology and/or treatment of schizophrenia, and that a combination of epigenetic and genetic approaches will be useful to understanding the molecular mechanism of this complex disease.

## Author contributions

Shusuke Numata and Barbara K. Lipska designed the experiments and contributed to the interpretation of the results; Shusuke Numata and Barbara K. Lipska performed experiments; Shusuke Numata, Barbara K. Lipska, and Tianzhang Ye analyzed the data; Shusuke Numata, Mary Herman, and Barbara K. Lipska wrote the paper.

### Conflict of interest statement

The authors declare that the research was conducted in the absence of any commercial or financial relationships that could be construed as a potential conflict of interest.
